# GeneFisher-P: variations of GeneFisher as processes in Bio-jETI

**DOI:** 10.1186/1471-2105-9-S4-S13

**Published:** 2008-04-25

**Authors:** Anna-Lena Lamprecht, Tiziana Margaria, Bernhard Steffen, Alexander Sczyrba, Sven Hartmeier, Robert Giegerich

**Affiliations:** 1 Dortmund University of Technology, Chair of Programming Systems, Dortmund D-44227, Germany; 2 Potsdam University, Chair of Service and Software Engineering, Potsdam D-14482, Germany; 3 Bielefeld University, Faculty of Technology, Bielefeld D-33594, Germany

## Abstract

**Background:**

PCR primer design is an everyday, but not trivial task requiring state-of-the-art software. We describe the popular tool GeneFisher and explain its recent restructuring using workflow techniques. We apply a service-oriented approach to model and implement *GeneFisher-P*, a process-based version of the GeneFisher web application, as a part of the Bio-jETI platform for service modeling and execution. We show how to introduce a flexible process layer to meet the growing demand for improved user-friendliness and flexibility.

**Results:**

Within Bio-jETI, we model the process using the jABC framework, a mature model-driven, service-oriented process definition platform. We encapsulate remote legacy tools and integrate web services using jETI, an extension of the jABC for seamless integration of remote resources as basic services, ready to be used in the process. Some of the basic services used by GeneFisher are in fact already provided as individual web services at BiBiServ and can be directly accessed. Others are legacy programs, and are made available to Bio-jETI via the jETI technology.

The full power of service-based process orientation is required when more bioinformatics tools, available as web services or via jETI, lead to easy extensions or variations of the basic process. This concerns for instance variations of data retrieval or alignment tools as provided by the European Bioinformatics Institute (EBI).

**Conclusions:**

The resulting service- and process-oriented GeneFisher-P demonstrates how basic services from heterogeneous sources can be easily orchestrated in the Bio-jETI platform and lead to a flexible family of specialized processes tailored to specific tasks.

## Background

The polymerase chain reaction (PCR) [1] is one of the most widely used techniques in biological laboratories today. It allows for isolating and exponentially amplifying fragments from a DNA sequence. Two short oligonucleotides (primers) are synthesized so that they can bind correctly at the 5' and 3' ends of the DNA region of interest. The PCR usually consists of a series of cycles (approx. 30) and is essentially carried out in three steps: (1) the denaturation step melts the double stranded DNA (94-96° C) into two separate strands. (2) During the annealing step the primers bind to the single-stranded template. Here, the temperature depends on the melting temperature of the primers. (3) In the extension step, a DNA polymerase synthesizes new DNA strands complementary to the single-stranded templates. This procedure is then repeated, doubling at each step the number of copies of the desired DNA segment. Through such repetitive cycles it is possible to obtain millions of copies of the desired DNA segment within a short period of time.

### PCR primer design with GeneFisher

**Figure 1:**
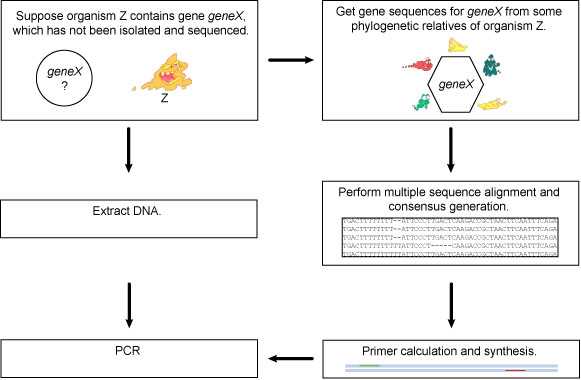
**Gene Fishing**. To amplify a gene X of organism Z, which DNA sequence is unknown, sequences of gene X are retrieved from phylogenetically related organisms. After alignment and consensus sequence calculation, primers are synthesized for the PCR reaction using Z's DNA.

Before each PCR experiment, specific primers have to be designed according to a number of criteria. Based on the assumption that genes with related function from different organisms show high sequence similarity, degenerate primers can be designed from sequences of homologous genes. *GeneFisher *[2,3] is a sophisticated interactive program for designing degenerate primers. The procedure leads to isolation of genes in a target organism using multiple alignments of related genes from different organisms. The term “gene fishing” refers to the technique where PCR is used to isolate a postulated but unknown target sequence from a pool of DNA (see figure 1).

For that purpose, GeneFisher selects PCR primers with certain criteria such as: melting temperature *T_m_*, GC content, primer length, 3' clamp GC content and degeneration, hairpin loop structure detection, primer-primer dimers detection, primer degeneration, amplified region length, and primer uniqueness. GeneFisher gives biologists the possibility of adjusting these parameters meeting the conditions of the PCR experiment.

**Figure 2:**
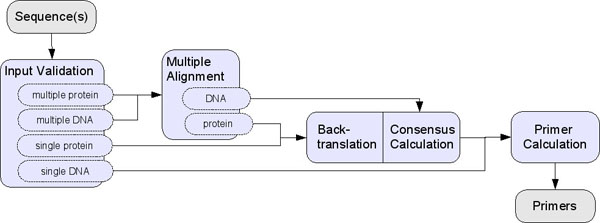
**GeneFisher Logical Process**. The GeneFisher logical process describes how the different kinds of inputs are processed before the actual primer calculation can take place. (Diagram following [6].)

GeneFisher accepts single or multiple DNA and protein sequences as input (see figure 2). As primers are calculated for a single DNA sequence, multiple input sequences are aligned using alignment programs such as ClustalW [4] or DCA [5]. From the alignment, a consensus sequences is derived and used as input for the primer calculation step. If protein sequences have been submitted, a backtranslation step is necessary, where amino acid sequences are translated to a hypothetical nucleotide sequence using either maximum redundancy methods or the codon usage of a given organism.

### GeneFisher implementation

The original GeneFisher implementation is a successful (more than 50,000 uses per year) but aged monolithic web application. It reveals a number of shortcomings, especially regarding user-friendliness and process monitoring/guidance. For instance, pauses in the workflow occur after requests to the server, and users have to check the state of their server requests and trigger the continuation of the application's execution manually. The monolithic nature of the code is a clear limitation.

### GeneFisher2

**Figure 3:**
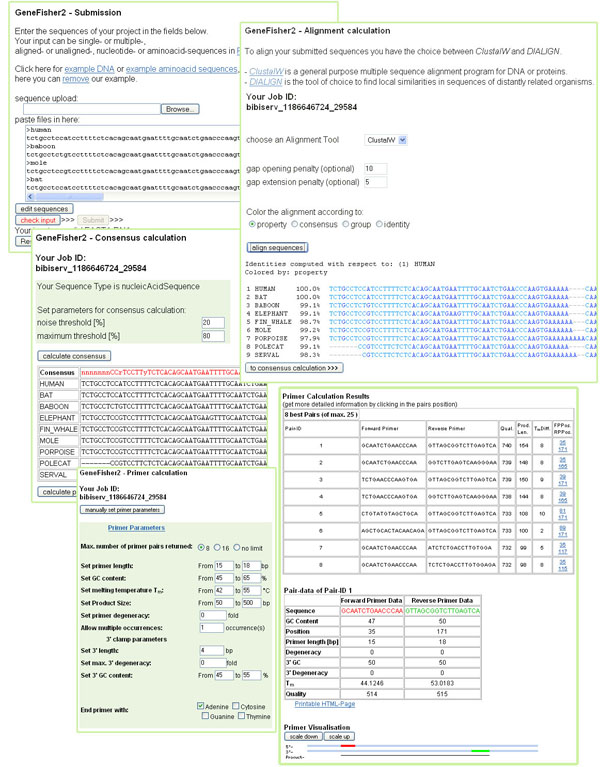
**Screenshots of the GeneFisher2 Web interface.** After sequence submission the user chooses an alignment tool and the parameters. Before consensus generation the alignment can be inspected and re-run with adjustments, if necessary. After consensus generation, primers are calculated. If GeneFisher cannot calculate any primers for given parameters, a rejection statistics suggests which parameters the user should change (not shown here).

GeneFisher2 [6] is a recent reimplementation of the original GeneFisher application which recreates the overall functionality of its predecessor while enhancing usability and user experience. This new version accesses basically the same underlying tools, now turned into components, via a web application, and conducts the whole application from a Web GUI by means of AJAX (Asynchronous Java-Script and XML) technology (see figure 3). GeneFisher2 uses this technology to connect to services hosted on the Bielefeld University Bioinformatics Server (BiBiServ) [7].

BiBiServ currently offers a variety of about 40 different software tools, spanning a wide area of applications such as Genome Comparison, Alignment methods (e.g. DCA or ClustalW), structure analysis (e.g. RNAshapes [8]), miRNA target prediction (e.g. RNAhybrid [9]) and PCR Primer Design (GeneFisher [2]). The tools are usually offered (amongst other access methods) as SOAP web services, allowing their usage as independent components in different scenarios. GeneFisher2 utilizes this component potential to create a much more flexible implementation of the general GeneFisher methodology.

The web service versions of the tools involved (DCA, ClustalW) are combined on the client-side, additionally employing the BioDOM web service [10] and HOBIT XML Data formats [11] for data conversion and validation. Due to this new program structure, several parts of the process can be modified and improved without breaking the whole system. It is, e.g., possible to use different alignment tools in the process by just adding the respective web services to the client-side JavaScript code, using the BioDOM web service to maintain consistency of input- and output data formats.

Using AJAX technology makes the GeneFisher2 web interface much more responsive than the old implementation. The integration of web services allows the usage of already implemented tools on the local or a remote server. But still, primer design remains a highly interactive process in GeneFisher2 and designing tens or hundreds of primers cannot be automated this way.

### GeneFisher-P

A new approach to provide GeneFisher's functionality in a convenient way is its transformation into a complex service that orchestrates the basic services and introduces a dedicated layer for processes.

**Figure 4:**
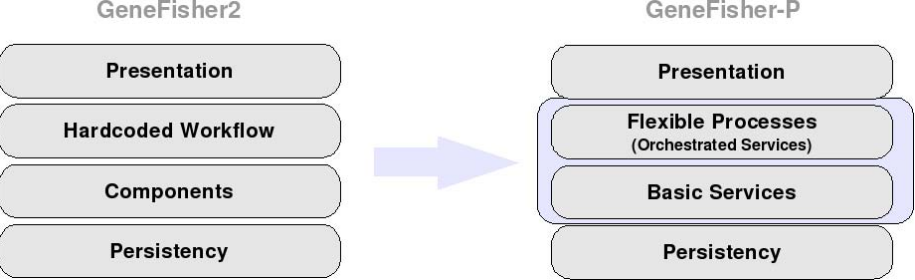
**GeneFisher2 vs. GeneFisher-P: Architecture Layers.** The GeneFisher2 architecture is a state-of-the-art component based system, while GeneFisher-P is a service-oriented realization, where processes form a flexible layer that decouples basic services from the GUI. The process layer is now accessible to the user for variations and modification.

*GeneFisher-P* puts the processes in the foreground. Following the design principles described in [12], it makes GeneFisher2's internally hidden processes explicit and accessible to the user. These processes are expressed in terms of a complex, reconfigurable business logic that uses (technically, orchestrates) an extensible collection of heterogeneous basic services. As shown in figure 4 (right), it separates the process modeling layer from the basic service layer and the GUI layer, in order to support process-oriented application repurposing, along the lines sketched in [13].

In particular, in GeneFisher2, a completely predefined application, the workflow is hidden from the end user, who can only interact with it through the web GUI. GeneFisher-P exposes the internal processes and the underlying services and components to the end user, who is now able to intervene and change or integrate them with others at ease.

In the following, we show how to turn a component-based application like GeneFisher2 into a collection of orchestrated composite services that implement sophisticated processes. To this aim, we first show how to integrate GeneFisher2's components into the *Java Application Building Center (jABC)* as basic services, then we remodel the original GeneFisher2 workflow as orchestrated process, show an execution example, and finally we discuss variations of GeneFisher-P, i.e., how to flexibly vary and extend the process into a family of task-specific processes by modifying the control structure or adding heterogeneous functionality to the process at the process model level.

## Results

The models of the GeneFisher-P process and its variations in Bio-jETI are realized as Service Logic Graphs in the jABC modeling framework. The jABC [12,14,15] is a general-purpose modeling framework which has already been successfully used to model and implement bioinformatics workflows [16,17]. In particular, it is a mature service engineering environment [18] that follows the Aggressive Model-Driven Development (AMDD) paradigm [19]. In contrast to the data-flow approach taken by many popular bioinformatics workflow systems like Taverna [20], models in the jABC are directed graphs that express the control-flow of a process. Currently, we are extending and improving our previous experience in the bioinformatics application domain in order to set up a comprehensive web-based service provisioning platform called Bio-jETI [21,22].

Basic services are called *SIBs* (Service Independent Building Blocks) in the jABC. SIBs use Java to encapsulate the functionalities from which whole processes can be composed at the process level. The process layer of an application thus becomes a true service orchestration or choreography, depending on whether the basic services are provided locally or in a distributed fashion. Access to local services is possible just as well as to remote tools, e.g. bioinformatics web services.

### Integrating GeneFisher2's components

According to GeneFisher2's internal logical workflow (figure 2), GeneFisher2 uses tools for input validation, multiple alignment, backtranslation, consensus calculation and primer calculation. Tools for these tasks are already available at BiBiServ [7]), where the original GeneFisher is provided as well. We need to make these tools available to jABC process modellers as libraries of basic services. In other words, SIBs are needed which provide access to these tools via the internet. Depending on the nature of these tools (here we have a mix of web services, legacy programs, and local activities), different technologies are used.

We discuss now how to deal with each of the three kinds of components: web services, legacy tools, and local components.

#### Web services

Some resources used by GeneFisher already offer web service interfaces and can thus be accessed via SOAP calls as customary:

• ClustalW [4,23] computes multiple alignments for DNA or protein sequences. The result is a biologically meaningful alignment of divergent sequences.

• DCA[5,24] stands for Divide-and-Conquer Multiple Sequence Alignment. This program is very fast and produces high quality multiple sequence alignments of protein, RNA, or DNA sequences.

In the GeneFisher web applications, the user can choose between these two tools for the multiple alignment. As we will show later, in the process-oriented approach such alignment components can be easily exchanged.

Furthermore, as in GeneFisher2, we use the BioDOM web service for the input validation: Input sequences for the web services at BiBiServ can be in plain text or, preferentially, in SequenceML [25] format. Starting with FASTA sequences (as, e.g., obtained from a database), these are converted into SequenceML via the SequenceMLfromFasta operation of the BioDOM web service [26].

If the conversion succeeds, the input fulfills the FASTA format specification and can be processed further, otherwise the process is aborted. Additionally, the types and numbers of sequences have to be figured out. In this version of the process we let the user provide this information, but the use of appropriate services would also be possible.

We use the BioDOM web service again when converting the XML-based sequence formats back to plain FASTA, the required input format for the legacy tools described next.

#### Legacy programs

Some of the required tools are already available from the original GeneFisher project, but are not directly accessible via the internet:

• BatCons performs the backtranslation (in case of protein sequences) and the consensus calculation (if the input consists of multiple sequences).

• gf_2000 is responsible for the actual primer design. The input has to be a single nucleotide sequence.

These legacy programs are written in C, compiled for and thus dependent on a particular CPU type.

They are integrated into the jABC as remote SIBs with the aid of the *Java Electronic Tool Integration platform (jETI)*[27,28]. To this aim, a jETI Toolserver is installed on the server machine. On the one side, the jETI Toolserver can access BatCons and gf_2000 via command line, on the other side it provides an interface to the internet. At runtime, it will accept service requests for these tools from the web and forward them to the actual tools, then collect the results and build adequate response messages. The actual call used by the jETI server to execute the tool and relevant request parameters are defined via the jETI Tool Configurator (accessible in a browser, also remotely). With this configuration information, SIBs can be automatically generated, downloaded, and then directly be used as representatives for the remote services in the jABC process models.

#### Local activities

In order to define the control flow and to achieve user interaction, we use some native jABC SIBs encapsulating local functionalities. For the control flow, these are mainly SIBs declaring variables or checking conditions, as well as for steering the flow of control in dependence of intermediate results. User interaction refers, e.g., to SIBs for file management and display of (intermediate) results. These functions are provided in the jABC environment as libraries of helper-SIBs, and are thus directly part of Bio-jETI.

### Modeling the GeneFisher process

The basic services introduced in the previous section, embodying local and remote functionalities, are sufficient to build a process model that reproduces GeneFisher's functionality. Based on SIBs encapsulating these services, we easily compose a process model that corresponds to the abstract workflow of figure 2 but provides much more information and is immediately executable.

**Figure 5:**
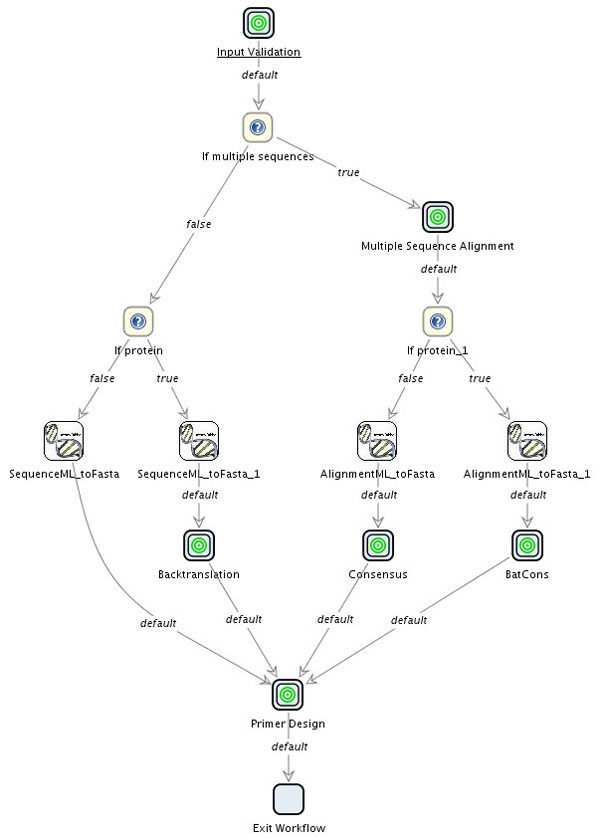
**Main GeneFisher Process in Bio-jETI**. According to the GeneFisher logical process, a jABC process can be orchestrated from SIBs integrating the BiBiServ services and local functionalities. The main GeneFisher process manages the control-flow according to the kind and number of input sequences. The input validation, multiple alignment, backtranslation, consensus calculation and primer design steps have been encapsulated into subprocesses.

We follow the main process, reported in figure 5, together with some of its subprocesses.

**Figure 6:**
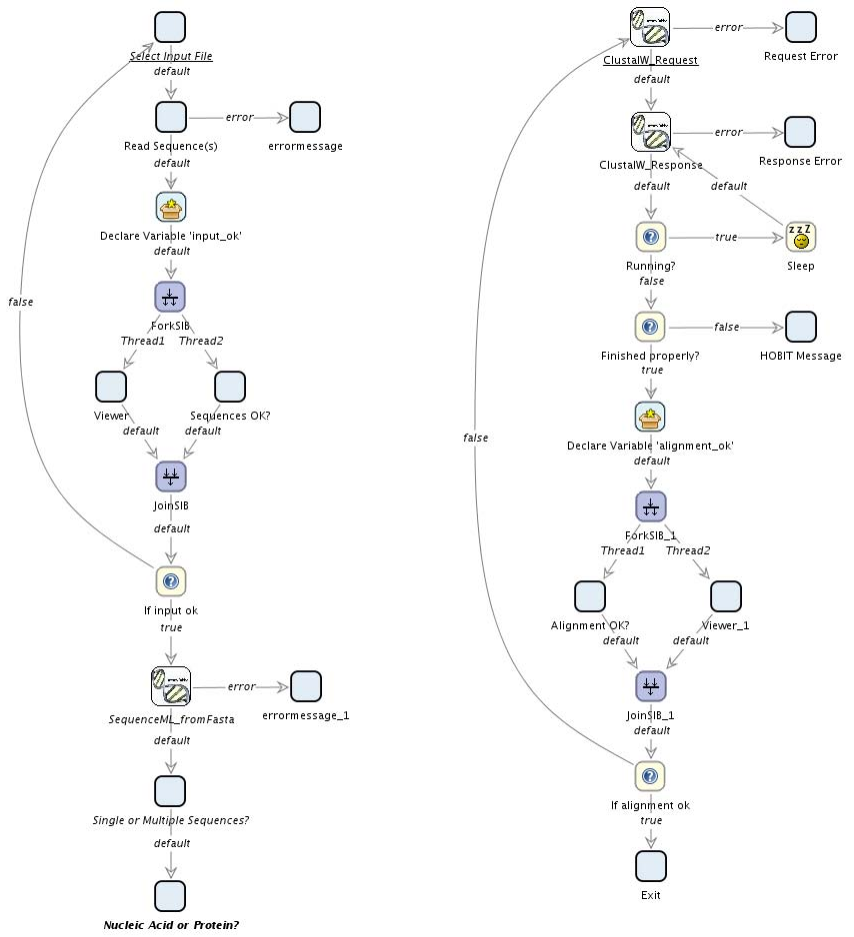
**Some GeneFisher Subprocesses in Bio-jETI.** The subprocesses for input validation (validating the input and determining kind and number of the sequences) and multiple sequence alignment (using BiBiServ's ClustalW web service). Like all subprocesses in GeneFisher-P (the remaining are not displayed), the intermediate results are displayed to the user in order to be approved or rejected before the execution proceeds.

• The process starts with the input validation, which has been encapsulated into a separate subprocess (see figure 6, left) for means of clarity. The jABC therefor offers full hierarchical modeling where subprocesses can be abstracted into so-called *GraphSIBs*, that enable viewing the process model at different levels of abstraction. Here the input file is read, checked by using the BioDOM web service as discussed earlier, and the number and type of the sequence(s) is entered by the user.

• When the input and its classifications are available, the necessary steps preceding the actual primer design are derived from this information by appropriate if-clauses. The SIBs If multiple sequences and If protein evaluate the variables mentioned before and this way steer the control flow according to the different preprocessing steps that are required for different inputs:

– *Single nucleotide sequences* (leftmost path) need no further processing before primer design.

– *Single protein sequences* (2nd path from left) have to be translated back into nucleic acids. They therefore go through the Backtranslation subprocess, not shown here in detail.

– *Multiple sequences* have to be aligned first (subprocess Multiple sequence alignment). Subsequently,

* for *Multiple nucleotide sequences* (2nd path from right) a consensus sequence has to be computed from the alignment in order to obtain a single sequence again.

* *Multiple protein sequences* (rightmost path) have to translated back into nucleic acids before the consensus calculation (subprocess BatCons).

• The multiple sequence alignment (encapsulated into an appropriate subprocess, see figure 6, right) is done asynchronously with the ClustalW web service here (BiBiServ's DCA can be used analogously). The required operations are request and response. Correspondingly the SIB ClustalW_Request submits the sequences and initiates the alignment computation, and ClustalW_Response is checked within a loop on the basis of the jobid that is available in the context after the initiation. While the job is running, the response will be a corresponding status, when it's finished, it contains the actual alignment.

• The backtranslations and consensus calculations are performed by a jETI-SIB that invokes the remote BatCons service.

• Finally, the single nucleotide sequence available at this point is submitted to the gf_2000 program via the appropriate jETI-SIB, which returns a set of possible primers. The results are then displayed by an appropriate local SIB.

Given the interactive nature of the GeneFisher web application, intermediate results are displayed to the user and may be accepted or rejected on demand. For example, the SIB Sequence OK? in the input validation subprocess asks the user if the data about to be submitted is what he intended to submit, and Alignment OK? asks if the alignment that is going to be used for the consensus calculation is satisfactory. In case of rejection, the process leads the control-flow back to a previous process step, typically to the SIB where the appropriate data or parameters can be modified before part of the process is executed again.

If errors occur during any task, an error message is written into the process context (which represents the session context for the orchestrated services) and can be displayed by the ErrorMessage SIB. This SIB is included at those points in the process where it is reasonable to provide a detailed error description, for example at the execution of remote services.

### Enacting the process: an execution example

jABC process models are immediately executable by the appropriate interpreter, the *Tracer* plugin, which is at the same time an execution environment. The Tracer allows for the stepwise or complete execution of the process, with additional capabilities characteristic of debugging tools for distributed executions, like using breakpoints, jumping into subprocesses or executing them atomically in one step, or following and advancing single threads singularly.

**Figure 7:**
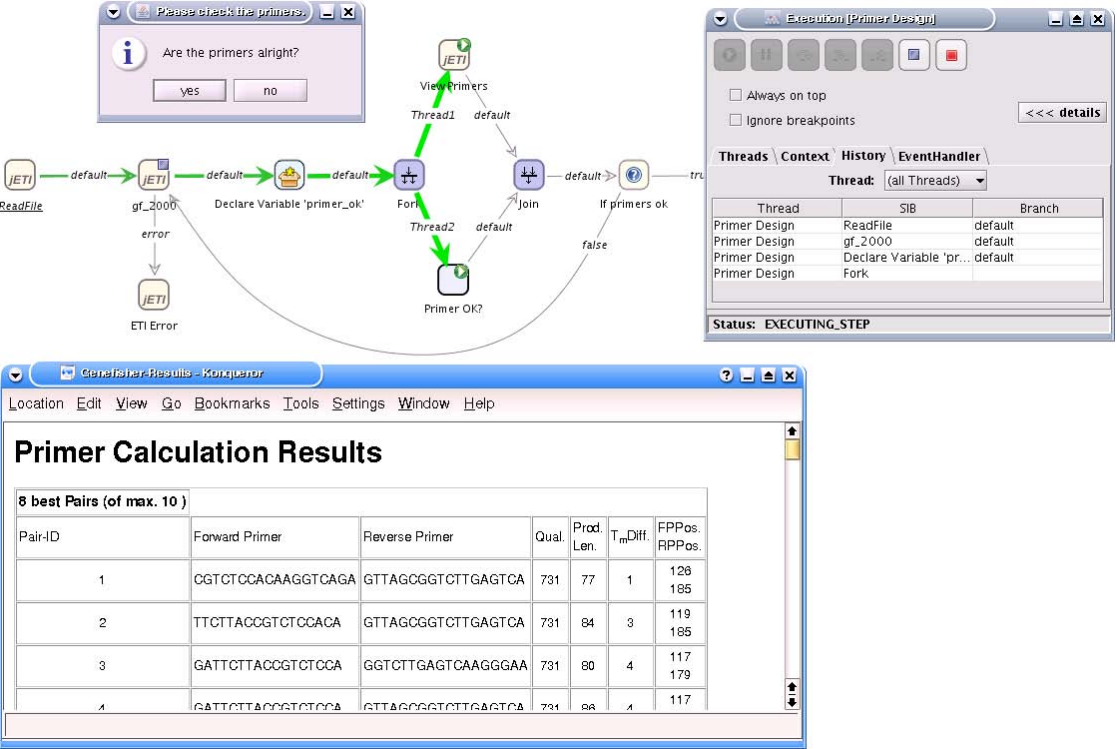
**Execution Example**. The Tracer plugin of the jABC makes it possible to directly execute the model. The figure shows the execution of the primer design subprocess, more precisely the step were the computed primers (here for the standard example data set from [3]) is displayed to the user in order to be approved or rejected.

Figure 7 shows part of the model at execution time. The Tracer provides detailed information about the execution, for example the variables and values that are available in the execution context or, in the figure, a history tab documenting the path through the model that has been taken in the current run.

### Dealing with process extensions and variations

Once the processes are available as executable models, they can be reconfigured and extended very easily, at the level of the service orchestration graphs, without need of programming.

As soon as the explicit formulation of the basic process models was available, we immediately had two proposals for variations, concerning an extended data retrieval feature and the use of an alternative sequence alignment service based on EBI services, which are external to BiBiserv.

The EBI provides, in fact, various web services, for example for database access and sequence analyses [29-31]. Meanwhile, SIBs are already available for accessing most of them from previous projects, and others can be automatically imported via the WSDL2SIB plugin also available in Bio-jETI.

**Figure 8:**
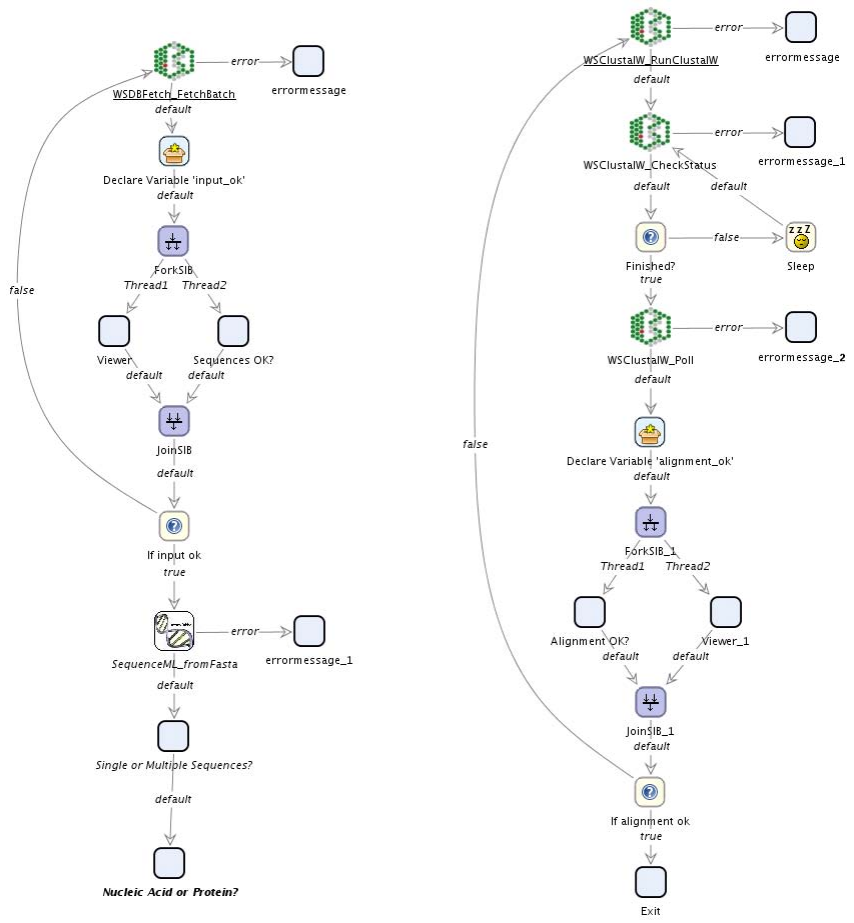
**GeneFisher-P Process with Extensions and Variations**. SIBs from other collections can be used to extend the process. For instance, the input sequences can be automatically retrieved from a database using the EBI's DBFetch web service (adapted input validation subprocess on the left), or specific services can be replaced with others, here the BiBiServ alignment tool with correspondent tools from the EBI (figure's right). The changes only take place in the concerned subprocesses, the main GeneFisher process is the same as in figure 5.

Thus it is possible to extend the previously presented workflow by automated data retrieval, or vary it using alternative tools for the multiple alignment (see figure 8) completely at the process level, inside the jABC.

#### Data retrieval with EBI's WSDBFetch

The WSDBFetch web service [32] allows for the retrieval of entries from various biological databases, such as EMBL for nucleotide sequences or UniProt for protein sequences. The sequences are identified by their IDs or accession numbers and can be retrieved in different formats, for instance FASTA. With the operation fetchBatch the web service provides the means for fetching a set of entries from a database by specifying their IDs.

Accordingly, we can start the process with the SIB WSDBFetch_FetchBatch before entering the bare GeneFisher workflow from figure 6, in order to retrieve a set of data from a database rather than using data from the local file system.

#### Multiple alignment with EBI's WSClustalW

Similar to BiBiServ, the EBI provides several web services for multiple sequence alignment. Like all analysis web services at the EBI, they provide distinct basic operations for running the application, checking the status and obtaining the results. Thus, SIBs are available for each of these operations and can be assembled in an own small process in order to create a correct asynchronous remote execution.

In case of WSClustalW [33], the required operations are runClustalW, checkStatus, and poll. Correspondingly, as shown in figure 8 (right), the SIB WSClustalW_RunClustalW submits the sequences and initiates the alignment computation, WSClustalW_CheckStatus is checked within a loop on the basis of the jobid that is available in the context after the initiation. When the job is finished, WSClustal_WPoll retrieves the actual alignment.

#### Further possible variations

The previous examples described how the process can be varied by carrying out particular execution steps by alternative tools. Even more sophisticated adaptions can be realized just as easy. For instance, it is possible to reduce the level of interactivity and run the primer design process in a loop in order to design large numbers of primers. It has been mentioned before that this is not feasible when carried out completely interactive. In Bio-jETI the model parts realizing any kind of interactivity can be excluded by simply directing the branches defining the control flow around them. For the repetition of the primer design process, the whole model can be transferred into a loop by adding a backward branch and a SIB working off a whole collection of inputs.

### Classifying and browsing the service libraries

Using the *TaxonomyEditor* plugin we can easily produce special-purpose classifications of the service libraries, called taxonomies, that support specific classification criteria or viewpoints. Taxonomies group services, and can be seen as very simple forms of ontologies, namely with a built-in is-a relation (subset concepts). In absence of any taxonomy, the jABC presents the available services in terms of the SIBs, and sorts them according to their location (as Java classes) in the filesystem. With the TaxonomyEditor users can arrange them according to any useful criteria, such as function (e.g. sequence aligners), origin (e.g. BiBiServ), technology (e.g web services) or input/output behaviour (e.g. string transformer).

For the above mentioned groups of service we set up two taxonomies: by their *provider* (i.e. BiBiServ or EBI), and by their *functionality* (e.g. multiple sequences alignment). Thus, when we intend to modify GeneFisher, but remain close to the original process, we can use the provider-based taxonomy in order to easily get the BiBiServ's services. Conversely, when we intend to replace certain components by equivalent ones (like discussed for the multiple alignment), the functionality-based taxonomy should be used.

### Workflow verification and code generation

The pure modeling could have been likely carried out in a different flavour also in other workflow tools, like Kepler and Taverna, but other functionality that Bio-jETI inherits from the underlying jABC framework add value to the pure modeling and execution.

#### Verification

During the model development process itself, different kinds of verification support can be used. The *LocalChecker* provides means to specify properties, preconditions, and environment conditions at the single component level. Examples of local check properties are the correct setting of parameters, the well-formedness of some inputs, the correct and complete connection of a SIB in the surrounding model (e.g., no dangling mandatory exits for a SIB) which would hamper the correct execution of the control flow.

More interestingly, we can also check global properties of a process. This is done with the *GEAR model checker*, a jABC plugin, which enables the model-wide verification of modal and temporal specifications expressed in CTL (Computation Tree Logic) or the μ-calculus [34]. Examples of such properties are

The input is not submitted to any remote service before it has been checked

or properties that describe what is legal for good runs, e.g. inside control loops, like

*It is possible to select a new input*.

*It is possible to re-do the alignment, the backtranslation, the consensus calculation, the primer design*.

The central issue here is that these are not local properties of a single service or processor, but properties of the process runs. They can be checked at process modeling time, *without execution* of the process. Therefore they offer a clear advantage over usual debugging methods.

#### Code generation

With the *GeneSys* code generation plugin users can automatically compile the whole process model into a single application, for example in Java. The generated application is a standard Java program or a servlet, which can be deployed on any platform for which a Java virtual machine is available (nowadays ranging from mobile phones to supercomputers).

The process execution is this way completely independent of Bio-jETI and of the jABC. This solves in particular also potential performance problems: the interpretation overhead is eliminated.

The GeneSys code generator can also produce C/C++, BPEL, and LeJos (the target language for LEGO Mindstorms robots) code, and it is currently being extended to cover other target languages (bioperl and C#).

## Discussion

Several workflow environments are already available for bioinformatics applications, with different characteristics. A popular platform is the Taverna workbench [20,35], which can integrate bioinformatics web services and some other kinds of remotely available tools. Other examples are the BioSPICE Dashboard [36], now discontinued as project, which integrates bioinformatics tools from different sources, or the WebLab [37], an internet platform providing web access to popular bioinformatics service programs.

In contrast to these frameworks, the Bio-jETI platform is based on the sound service engineering techniques of the underlying the domain-independent jABC modeling framework. The full power of model-driven design is maintained when adapting the framework for the biology domain.

Comparing Bio-jETI to Taverna, the basic difference is that Taverna's workflows describe the data-flow, while models in the jABC define the control-flow of a process. Both approaches are usually considered to be capable of expressing the same processes, but in fact there are limitations with respect to the inclusion of elaborate control structures when using the data-flow approach: the Taverna workbench offers control links in addition to the basic data links, but these are merely sufficient to express simple dependencies.

In the jABC a set of different common control structures is already available and shared among its different application domains. They allow for the modeling of sophisticated processes, for instance with different execution traces depending on the kind of input data, as shown above, or with iterations or recursions over sets of data. The data itself is managed within an *execution context* of the model, and identifiers similar to variables can be used to refer to particular data items.

Both Bio-jETI and Taverna support graphical modeling: they provide distinct canvas for the palette of available components (called *processors* in Taverna, *SIBs* in the jABC), the configuration of the components and the whole model, and for the display of the model itself. However, while in Taverna the design is entirely done via context menus, and from this information a read-only graphical representation of the workflow is generated, in Bio-jETI the model is constructed in a convenient and intuitive way by dragging and dropping and configuring the selected components on the canvas.

Both frameworks provide means for the direct enactment of the model and for the integration of web services on the basis of their WSDL descriptions. Taverna even performs an automated service discovery at startup. While Taverna can additionally invoke *Soaplab* and *Talisman* services, Bio-jETI integrates almost arbitrary tools via the ETI technology: command-line, WSDL, CORBA IDL and REST imports are already provided with the platform.

The additional benefits offered by the jABC plugins for verification and code generation are so far unique to Bio-jETI.

## Conclusions

We have proved in practice on a well known tool (GeneFisher) that remodeling its processes in Bio-jETI is a swift and easy business, and that making those processes explicit and first-class citizens of a distinct architectural layer pays off in terms of enhanced flexibility and ease of modification and variation. Our view of basic services, the SIBs, provides a uniform interface to heterogeneous sources and can be assembled and exchanged easily, this way achieving at the same time a high level of modeling flexibility and executability of the modeled processes.

The additional capabilities to verify the conformance of the designed processes with respect to desirable properties and the code generation offered by Bio-jETI are object of ongoing work.

## List of abbreviations used

AJAX: Asynchronous Java and XML

AMDD: Aggressive Model-Driven Design

BiBiServ: Bielefeld University Bioinformatics Server

BPEL: Business Process Execution Language

CORBA: Common Object Request Broker Architecture

CPU: Central Processing Unit

CTL: Computation Tree Logic

DCA: Divide-and-Conquer Alignment

DNA: Deoxyribonucleic Acid

DOM: Document Object Model

EBI: European Bioinformatics Institute

GEAR: Game-Based Easy And Reverse Model-Checking Tool

GUI: Graphical User Interface

HOBIT: Helmholtz Open Bioinformatics Technology

IDL: Interface Description Language

jABC: Java Application Building Center

jETI: Java Electronic Tool Integration Platform

PCR: Polymerase Chain Reaction

REST: Representational State Transfer

RNA: Ribonucleic Acid

SequenceML: Sequence Markup Language

SIB: Service-Independent Building Block

WSDL: Web Service Description Language

XML: Extensible Markup Language

## Competing interests

The authors declare that they have no competing interests.

## Authors' contributions

AL implemented or generated the basic services from the existing deployed components and tools, built the models and drafted the manuscript. TM and BS have been developing the concept of the jETI platform since 1997, first in the area of formal verification tools, then in the area of Semantic Web services. They lead the development of the bioinformatics application of the jABC. They have revised and edited the manuscript. SH, AS and RG provide and maintain the BiBiServ infrastructure, conceived GeneFisher2 and edited the part of the manuscript describing the traditional GeneFisher approach and tool. All authors read and approved the final manuscript.
